# Fabrication of Copper Sulfide Nanoparticles and Limonene Incorporated Pullulan/Carrageenan-Based Film with Improved Mechanical and Antibacterial Properties

**DOI:** 10.3390/polym12112665

**Published:** 2020-11-12

**Authors:** Swarup Roy, Jong-Whan Rhim

**Affiliations:** Department of Food and Nutrition, BioNanocomposite Research Institute, Kyung Hee University, 26 Kyungheedae-ro, Dongdaemun-gu, Seoul 02447, Korea; swaruproy2013@gmail.com

**Keywords:** pullulan/carrageenan, CuSNP, limonene, composite film, mechanical property, antibacterial activity

## Abstract

Edible biopolymer (pullulan/carrageenan) based functional composite films were fabricated by the addition of copper sulfide nanoparticles (CuSNP) and D-limonene (DL). The DL and CuSNP were compatible with the pullulan/carrageenan biopolymer matrix. The addition of CuSNP significantly increased the UV-blocking properties without substantially reducing the transparency of the film. The addition of CuSNP improved the film’s tensile strength by 10%; however, the DL addition did not significantly influence the strength, while the combined addition of CuSNP and DL increased the strength by 15%. The addition of the fillers did not significantly affect the thermal stability of the film, but the water vapor barrier property was slightly improved. There was no significant change in the moisture content and hydrophobicity of the composite film. Besides, the composite film showed some antimicrobial activity against food-borne pathogenic bacteria. The fabricated pullulan/carrageenan-based film with antimicrobial and UV-barrier properties is likely to be used in active food packaging applications.

## 1. Introduction

Today, the use of plastics is rapidly increasing not only in the food packaging sector, but also in all other industries, and they generate enormous plastic waste and environmental pollution due to their non-degradability [1,2]. Since the industrialization of plastics in the 1950s, its production has increased exponentially every year, and now around 400 million tons of plastic are produced annually worldwide [3]. This increase in production is expected to reach about 180 million tons in 2050 [4,5]. In Korea alone, the annual consumption of disposable plastics is more than 600,000 tons, and accordingly, plastic waste also increases significantly, generating about 3 million tons of plastic waste every year. Only 22.3% of these were managed by the extended producer responsibility [6]. In general, there are two ways to solve this problem: one is to reduce plastic waste and increase the recycling rate, and the other is to use biodegradable materials that can replace plastics. In this context, biopolymer plastics or bioplastics, which uses annually renewable resources, is an eco-friendly material that can replace petroleum-based plastics [7,8]. Carbohydrates are used to manufacture biopolymer packaging films due to their excellent film-forming ability, good gas barrier properties, and mechanical properties. Pullulan, a carbohydrate produced by microorganisms, is a homo-polysaccharide composed of repeating units of maltotriose. It is a highly water-soluble, colorless, and odorless substance. It has excellent gas and oil barrier properties, making it an ideal choice to make edible films and coatings material. The pullulan-based film has high hydrophilicity, low transparency, and poor mechanical properties [9]. The high cost is another problem with pullulan. One way to solve this problem is to make a composite film by blending pullulan with another compatible polymer such as carrageenan. Carrageenan is a linear sulfated polysaccharide extracted from seaweed, a cost-effective biopolymer known to produce transparent films with moderate mechanical and barrier properties, but the film is very brittle [10,11]. Combining the pullulan and carrageenan blend film is expected to be an excellent film that can improve physical properties and supplement each biopolymer’s problems. Previously, blending various biopolymers such as alginate, carboxymethyl cellulose, chitosan, and casein has been tested to improve the physical properties of pullulan film [9,12,13,14].

The functional properties of composite films are also essential in active and intelligent food packaging applications. A functional pullulan/carrageenan film can be developed by adding functional materials such as nanofillers and bioactive compounds. The addition of nanofillers and bioactive compounds is expected to improve the film’s physical and functional properties.

Recently, copper sulfide nanoparticles (CuSNP) have emerged as a potential nanofiller that enhances films’ physical and functional properties [15,16]. CuSNP has received considerable attention due to its low toxicity and potential applications in biological fields such as drug delivery, photothermal therapy, antimicrobial agents, in vitro bio-sensing, etc. [17,18,19,20,21]. Another potential bioactive functional compound is limonene, a monoterpene found primarily in citrus fruits such as lemons, grapefruits, oranges, etc. [22]. Limonene, belonging to the GRAS material, is also used in many applications such as flavorings, food preservatives, etc. [23,24]. Recently, some functional films reinforced with CuSNP have been reported [15,16,25], and there have been several reports of functional films with added limonene [22,26,27,28]. Considering the potential of CuSNP and limonene as functional materials, synergies can be expected when these materials are used together. To the best of our knowledge, so far, there are no reports of manufacturing pullulan/carrageenan-based functional films by combining CuSNP with D-limonene.

Therefore, the primary purpose of this study was to prepare a pullulan/carrageenan-based functional film by integrating CuSNP and limonene as fillers for active food packaging applications. The effect of CuSNP and limonene alone or in combination with pullulan/carrageenan-based films were closely investigated.

## 2. Materials and Methods

### 2.1. Materials

Pullulan powder was obtained from Korea Bio Polymer Co. Ltd. (Bucheon, Gyeonggi-do, Korea). Food grade carrageenan was purchased from MSC Co., Ltd. (Sungnam City, Gyeonggi-do, Korea). Glycerol was acquired from Daejung Chemicals & Metals Co., Ltd. (Siheung, Gyeonggi-do, Korea). D-limonene (DL) was purchased from Sigma-Aldrich (St. Louis, MO, USA). Brain heart infusion broth (BHI), tryptic soy broth (TSB), and agar powder were obtained from Duksan Pure Chemicals Co., Ltd. (Ansan, Gyeonggi-do, Korea). *Escherichia coli* O157: H7 ATCC 43895 and *Listeria monocytogenes* ATCC 15313 were procured from the Korean Collection for Type Culture (KCTC, Seoul, Korea). The copper sulfide nanoparticles (CuSNP) used in this study were prepared as previously reported [25].

### 2.2. Preparation of Films

**Scheme 1:**
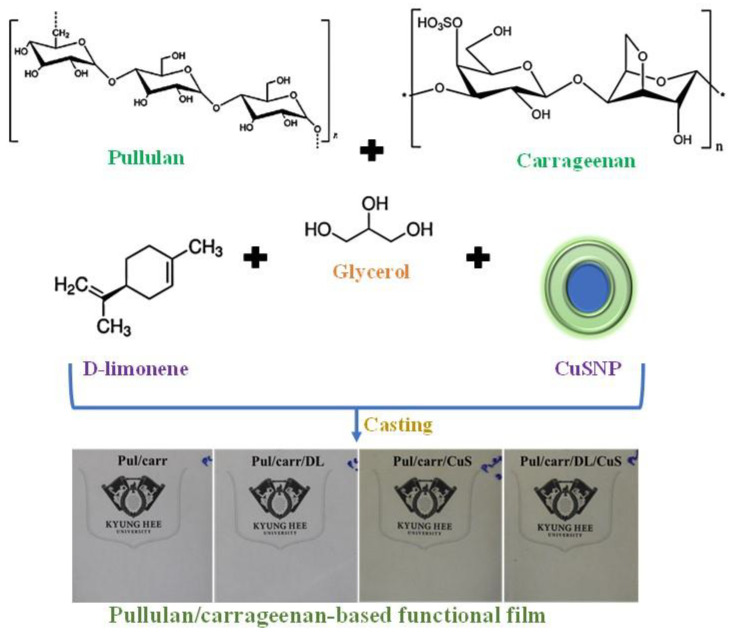
Schematic presentation for the preparation of Pul/Carr-based films.

The pullulan/carrageenan-based film was prepared using a solution casting method [29,30], as shown schematically in Scheme 1. For the preparation of film solution, DL (5 wt.% based on biopolymer) was mixed with 150 mL distilled water with vigorous mixing using a magnetic stirrer. The CuSNP (0.5 wt.% based on biopolymer) was dispersed in 150 mL of distilled water using a magnetic stirrer and then ultrasonicated at 60% amplitude for 3 min with pulses 5 s on and 2 s off in a probe ultrasonicator (Model VCX 750, Sonics & Materials, Inc., New Town, CT, USA). Mixed solutions of CuSNP (0.5 wt.%) and DL (5 wt.%) were also prepared. To the CuSNP and DL dispersed solutions, 1.2 g of glycerol (30 wt.% based on polymers) was added with continuous stirring. Then, 4 g of biopolymers (2 g each of pullulan and carrageenan) was dissolved slowly and heated for 20 min at 95 °C with constant stirring. The film-forming solution was cast on a flat Teflon film-coated glass plate and dried at room temperature for 48 h. The dried film was peeled from the container and conditioned at 25 °C and 50% RH for at least 48 h. For comparison, control pullulan/carrageenan was prepared following the same procedure without adding CuSNP and DL. The produced films were designated as Pul/Carr, Pul/Carr/DL, Pul/Carr/CuSNP, and Pul/Carr/DL/CuSNP depending on the type of biopolymer and filler material.

### 2.3. Characterization and Properties of the Film

#### 2.3.1. Optical Properties

The films’ surface color (*L*, *a*, and *b-*values) was evaluated using a Chroma meter (Konica Minolta, CR-400, Tokyo, Japan). The total color difference (∆*E*), whiteness index (*WI*), Chroma (C), and yellowness index (*YI*) of the film were calculated as follows:

(1) $$\Delta E=\sqrt{{\left(\Delta L\right)}^{2}+{\left(\Delta a\right)}^{2}+{\left(\Delta b\right)}^{2}}$$

where ∆*L*, ∆*a*, and ∆*b* are the difference between each color value of the standard color plate and film sample, respectively.

*YI* = (142.86 × *b*)/*L* (2)

(3) $$\mathrm{WI}=100-\sqrt{\left(100-L\right)+a^{2}+b^{2}}$$

(4) $$C=\sqrt{a^{2}+b^{2}}$$

The UV-vis spectra of the Pul/Carr-based film were recorded using a UV-vis spectrophotometer (Mecasys Optizen POP Series UV/Vis, Seoul, Korea), and the UV-barrier and transparency properties of the film were assessed by measuring the light transmittance at 280 (T_280_) and 660 nm (T_660_), respectively [16].

#### 2.3.2. Morphology, FTIR, and XRD

The film samples’ microscopic morphological view was inspected using a FESEM (FE-SEM, SU 8010, Hitachi Co., Ltd., Matsuda, Japan). FTIR spectra of the film samples were noted using an attenuated total reflectance-FTIR spectrophotometer (TENSOR 37 Spectrophotometer with OPUS 6.0 software, Billerica, MA, USA) at a wavenumber of 4000–500 cm^−1^ with the resolution of 32 scans at 4 cm^−1^. The XRD pattern of the film sample was determined at 2θ = 20–80° using an XRD diffractometer (PANalytical X’pert Pro MRD Diffractometer, Amsterdam, Netherlands) at a scan rate of 0.4°/min.

#### 2.3.3. Mechanical Properties

The film’s thickness was measured using a hand-held digital micrometer (Digimatic Micrometer, QuantuMike IP 65, Mitutoyo, Japan) with an accuracy of 1 μm. The film thickness was measured at five random locations of each film, and their average value was reported. Each film’s mechanical properties were determined according to the standard method of ASTM D 882-88 using an Instron Universal Testing Machine (Model 5565, Instron Engineering Corporation, Canton, MA, USA). For the measurement, the film sample was cut with (2.54 × 15 cm) dimension using a precision double blade cutter (model LB.02/A, Metrotec, S.A., San Sebastian, Spain) [31]. The machine was operated in the tensile mode with an initial grip separation and crosshead speed set at 50 mm and 50 mm/min, respectively.

#### 2.3.4. Water Vapor Permeability (WVP), Water Contact Angle (WCA), and Moisture Content (MC)

The WVP of the films was determined gravimetrically using the ASTM E96-95 standard method [16]. At first, the WVP cup was first filled with a prescribed amount of water and then covered with the film and sealed and kept in the controlled environmental chamber at 25 °C and 50% RH. After equilibration, the WVP cup’s weight was measured at every one-hour interval, and weight loss was calculated. The WVTR (g/m^2^ s) was determined from the slope (linear) of the steady-state portion of the weight loss vs. time curve. Then, the WVP of the films was calculated in g m/m^2^ Pa s as follows:

WVP = (WVTR × L)/Δp(5)

where L was the film thickness (m), and Δp was water vapor partial pressure difference (Pa) across the film.

The film’s surface wettability was determined by computing the film surface’s water contact angle using a WCA analyzer (Phoneix 150, Surface Electro Optics Co., Ltd., Kunpo, Gyeonggi-do, Korea). For the measurement, the film sample was fixed on the film holder, and then a drop of water (~10 μL) was added to the surface of the film and immediately determined the WCA.

The MC of the film was determined by following the previously published methodology [32]. The MC of the film samples was determined by checking the weight of the film sample (W_1_), and then the same film was dried in a hot air oven at 105 °C for 24 h and then weighed again (W_2_). The MC of the film was expressed as a percentage of the initial weight of the film:

(6) $$\mathrm{MC}\;\left(\%\right)=\frac{W_{2}-W_{1}}{W_{1}}\times 100$$

#### 2.3.5. Thermal Analysis

The films’ thermal stability was determined using a thermogravimetric analyzer (Hi-Res TGA 2950, TA Instrument, New Castle, DE, USA). For measurement, ~10 mg of film sample was taken in a standard aluminum pan and scanned at a heating rate of 10 °C/min in a temperature range of 30–600 °C under a nitrogen flow of 50 cm^3^/min with an empty pan as a reference [32].

### 2.4. Antibacterial Activity

The film specimen’s antibacterial activity was tested against food-borne pathogenic bacteria, *E. coli,* and *L. monocytogenes* by following a previously published method [33]. The microorganisms were inoculated in the TSB and BHI broth, respectively, and subsequently cultured overnight at 37 °C with agitation at 100 rpm. After diluting the inoculum appropriately, 100 μL of the diluted inoculum was transferred to 20 mL of TSB and BHI broth containing 100 mg of film samples and incubated at 37 °C for 12 h with agitation at 120 rpm. Samples were taken out at a predetermined time interval and plated on agar plates after appropriate dilution to evaluate the viable colonies. For calculation, an antibacterial test was performed using culture medium without film and neat film as negative control and positive control, correspondingly. The antimicrobial tests were done in triplicate.

### 2.5. Statistical Analysis

For statistical analysis of the obtained results, one-way analysis of variance (ANOVA) was performed, and the significance of each mean property value was determined (*p* < 0.05) by Duncan’s multiple range test using the SPSS statistical analysis computer program (SPSS, Inc., Chicago, IL, USA).

## 3. Results and Discussion

### 3.1. Apparent Color, Morphology, and Optical Properties

**Figure 1:**
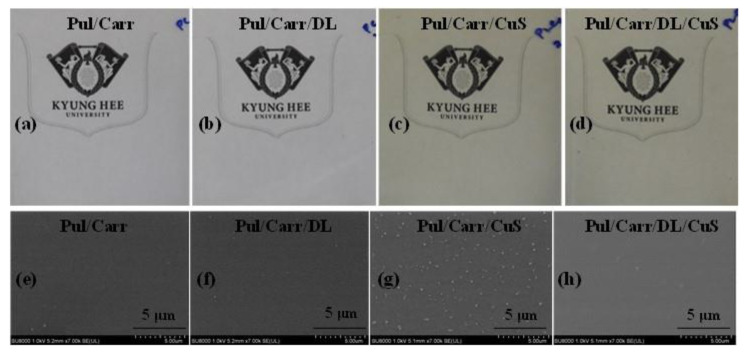
The visual appearance (**a**–**d**) and microstructure (**e**–**h**) of the pullulan/carrageenan-based films.

The macroscopic appearance of all the prepared films is displayed in Figure 1a–d. The Pul/Carr and Pul/Carr/DL films were transparent without color, but the CuSNP-added films were greenish. The films’ morphology was observed by the FESEM (Figure 1e–h), and it shows all the films were intact and without any apparent defects. The blending of carrageenan in pullulan makes a uniformly distributed homogenous and compatible film. The fillers were also evenly mixed in the matrix polymer, indicating their good miscibility in the liquid phase. In addition, there were no cracks or voids in the surface image and no apparent accumulation of particles, indicating both CuSNP and DL are compatible with the polymer matrix. The excellent compatibility was due to the strong adhesion, intermolecular binding, and affinity between the fillers and the matrix polymer.

**Figure 2:**
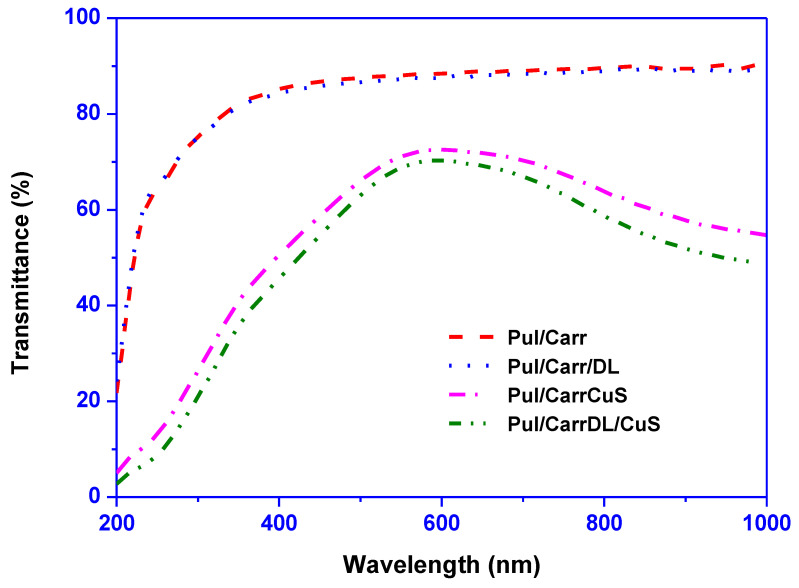
Light transmittance spectra of pullulan/carrageenan-based films.

UV-visible transmission spectra determined the film’s optical properties, and the results are displayed in Figure 2. The UV-vis spectra exhibit that the Pul/Carr and Pul/Carr/DL films were transparent. On the other hand, the CuSNP-added (alone or combined) composite film’s absorption profile was completely different due to the near-infrared absorption of CuSNP [15]. For further understanding, the film’s UV-light barrier and transparency were scrutinized by measuring the absorbance at 280 nm and 660 nm, respectively, and the results are displayed in Table 1. The UV light transmittance and transparency of the control film were 70.1% and 88.3%, respectively. The addition of DL did not much influence the optical properties but was greatly affected by the incorporation of CuSNP. The UV-light barrier property was increased ~75%, which is very significant, whereas the transparency remains ~70%, which is suitable for packaging application. The substantial increase in UV-light barrier property was due to the UV-light absorption of CuSNP [25]. CuSNP and DL’s present findings on Pull/Carr films’ color properties are consistent with the previously published results [16,26]. Therefore, the current finding suggested that the blending of CuSNP/DL improved the UV-light barrier properties of the film without greatly affecting the transparency.

Table 1 also shows the surface color and light transmittance properties of the films. The lightness (Hunter *L*-value) of the film was not significantly affected by the DL, whereas the addition of CuSNP slightly reduced the lightness. The lightness, *a*-value, *b*-value, and Δ*E* were not much changed for DL, whereas it decreased for the CuSNP-added film. The drastic changes in the color parameter were mainly due to the greenish color of CuSNP. The color variation of the CuSNP/DL-added Pul/Carr film was further perceived by analyzing the *C*, *WI*, and *YI* values. The obtained results are consistent with the Hunter color parameter. For DL-added film, there was no significant alteration in *C*, *WI*, and *YI* value. For the CuSNP-added film, the *WI* was decreased slightly, whereas the *C* increased similar to the Δ*E*. The *YI* of the film also increased significantly in the case of CuSNP-added film. Overall, the luminosity of the film was high, which could be useful for packaging applications. The effect of CuSNP on the color parameter was similar to the previously published data [16].

### 3.2. FTIR and XRD

**Figure 3:**
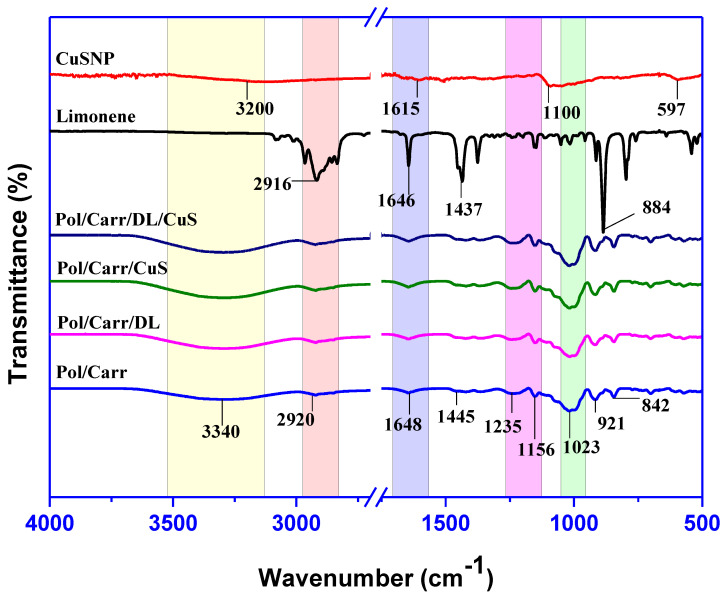
FTIR spectra of CuSNP, DL, and pullulan/carrageenan-based films.

The FTIR spectra of CuSNP, DL, and the pullulan/carrageenan-based films are shown in Figure 3. The characteristic peaks of limonene were obtained at 2918, 1645, 1436, and 885 cm^−1^ due to the –C–H stretching, –C=C stretching, –C–H bending, and out-of-plane bending, respectively [34]. In the case of CuSNP, peaks were seen at 3200, 1615, 1100, and 597 cm^−1^ due to the O–H stretching, C–O bending, –C–O stretching, and the pyranoid ring of corn starch capped CuSNP, respectively [35]. The broad peaks in the range of 3600–3000 cm^−^^1^ observed in all films were due to O–H stretching vibration and intermolecular or intramolecular H-bonding [36]. The peak at 2920 cm^−^^1^ was ascribed to the C–H stretching vibration of the methylene group of the matrix biopolymer chain [36]. The peak found at 1648 cm^−1^ was attributed to the amide-I of carrageenan [37]. A peak found at 1235 cm^−1^ was due to the presence of sulfate ester groups in carrageenan [38], and the peak noticed at 1156 cm^−1^ was ascribed to the stretching vibration of the (α-1-4) glycosidic bond of pullulan [39]. The peak at 1023 cm^−1^ corresponded to the C–O stretching bond of pullulan [12]. The peak seen at 921 cm^−1^ was due to 3, 6-anhydro-D-galactose of carrageenan, and the peak observed at 842 cm^−1^ was due to galactose-4-sulfate of carrageenan and α-glucopyranose units of pullulan [37,39]. The observed results indicate that the blending of carrageenan in pullulan makes a compatible composite film. In addition, the addition of filler did not change the chemical structure of neat biopolymers. The observed FTIR results conclude that, except for slight alteration in peak intensity, there was no significant alteration in the film’s functional groups, suggesting that the film’s chemical structure was not transformed after incorporating the filler.

**Figure 4:**
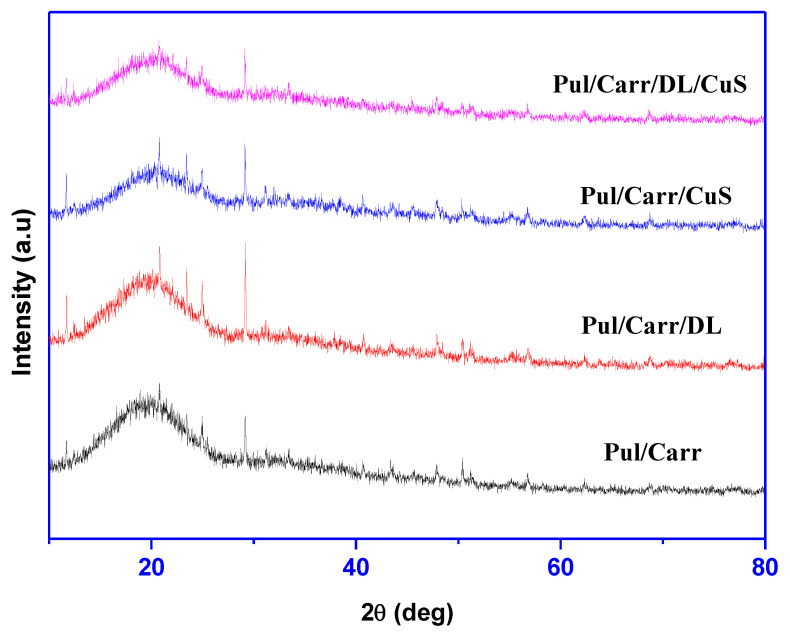
XRD pattern of pullulan/carrageenan-based films.

The XRD pattern of the Pul/Carr-based films are presented in Figure 4. All the tested films showed a characteristic broad peak ~20° and some unknown peaks, which is presumably due to the amorphous nature of the biopolymers (pullulan and carrageenan). Except for some slight alteration, the XRD data of the Pul/Carr-based composite films do not show any clear change in peak pattern after the addition of DL and CuSNP. Although CuSNP show characteristic XRD patterns [25], they did not appear in the present findings as only 0.5% of CuSNP was used.

### 3.3. Mechanical Properties

The thickness and mechanical properties of the films are presented in Table 2. The film’s thickness was ~47–49 μm and was not much altered after blending with the fillers. The addition of fillers meaningfully influenced the mechanical properties of the films (Table 2). The tensile strength (TS) of the film increased significantly by adding CuSNP alone or in combination, although it was not greatly affected by the addition of DL. The flexibility (EB) and stiffness (EM) of the CuSNP/DL incorporated film also increased meaningfully. The addition of only CuSNP improved the TS by 10%. The combined addition of CuSNP/DL improved 15% of the TS, probably due to the increase in molecular interaction between the matrix polymer and fillers and good interfacial surface interactions. Overall, the fillers’ addition in the Pul/carr-based film improved the film’s strength, flexibility, and stiffness. The CuSNP is already recognized as an efficient nanofiller for enhancing the mechanical properties of the biopolymer-based film. In the case of CuSNP-added agar, alginate, and carrageenan film, a similar improvement in mechanical properties was reported previously [15,16,25]. Similar to the current findings, the tensile strength of the LDPE film was found to not have changed significantly with the addition of lemon aroma [27]. On the other hand, it has been reported that the addition of limonene significantly reduces the mechanical properties of the PLA film [26]. Conversely, it has been reported that PVA/chitosan films have significantly improved mechanical properties by adding DL [22]. Overall, the addition of fillers improves the mechanical properties of the Pul/carr-based film.

### 3.4. WVP, WCA, and MC

The WVP of all the films is also presented in Table 2, and the results indicate the good water vapor barrier properties of the film (~1.0 × 10^−9^ g m/m^2^ Pa s). The WVP of the Pul/Carr-based film was also affected by the addition of filler. The effect was more significant in the combined addition of CuSNP/DL than only CuSNP or DL. The film’s increased water vapor barrier properties might be due to creating a tortuous water vapor diffusion path formed by the well-distributed water vapor impermeable filler [22]. Similar to this observation previously, it was also described that DL’s addition improved the water vapor barrier property of the PVA/chitosan film [22]. Earlier, it was explained that the addition of CuSNP at a lower content in alginate and agar-based films improved the water vapor barrier properties [16,25].

The WCA of the films is also shown in Table 3. The WCA of the control film and all other films were < 65°, representing their hydrophilic nature [40]. The incorporation of CuSNP and DL alone or in-combination into the film slightly increased the WCA, although the changes were statistically insignificant (*p* < 0.05). The increase in the WCA of the films was mainly due to the presence of CuSNP and DL, which are hydrophobic. Previously in the case of CuSNP-added agar and alginate film, an increase in the WCA was reported [16,25]. In contrast to the current observation in the case of DL-added PVA/chitosan film and PLA-based film, a significant increase in WCA was reported recently [26,22].

The moisture content of all the tested films was ~11–12%, which is relatively low and suitable for packaging film. The moisture content of the filler added film was not altered meaningfully compared to the control film. Similar MC results were reported in previously published pullulan-based packaging film recently [36].

### 3.5. Thermal Stability

**Figure 5:**
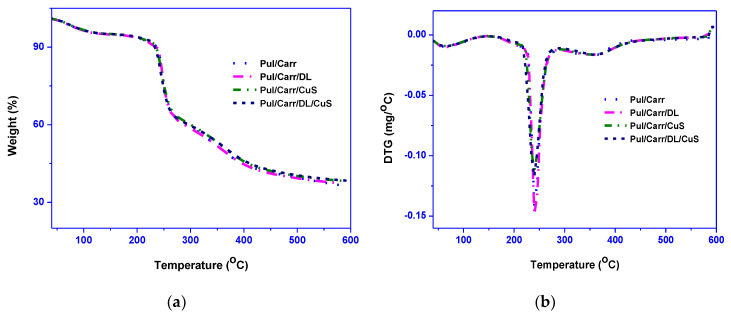
(**a**) TGA and (**b**) DTG thermograms of pullulan/carrageenan-based films.

The thermal stability (TGA/DTG) of the films are shown in Figure 5. During thermal decomposition, all the film showed a multi-step weight loss. The first weight loss occurred at 50−115 °C, mostly due to the film’s humidity. The second and main weight loss was observed at 160−280 °C with a maximum of around 240 °C due to glycerol and biopolymer degradation [38]. The final stage of weight loss occurred at 290−410 °C due to the remaining biopolymers’ decomposition [37,41]. The decomposition temperatures (onset/endset), 50% decomposition temperature (T_0.5_), and the films’ char content are shown in Table 3. The T_0.5_ of the CuSNP added film was increased ~10 °C, whereas it remains unchanged for DL. The maximum decomposition temperature of the film was not affected by the presence of fillers. Similar thermal stability results were reported in the case of DL-added PLA, and CuSNP-added alginate film previously [16,26]. TGA test results designated that the incorporation of fillers does not alter the Pul/carr-based film’s thermal stability. The char content at 600 °C for the film was varied in the range of 36–38% depending on the type of filler, and the relatively high char content of the films was due to the non-ignitable minerals present in the biopolymer [38].

### 3.6. Antimicrobial Activity

**Figure 6:**
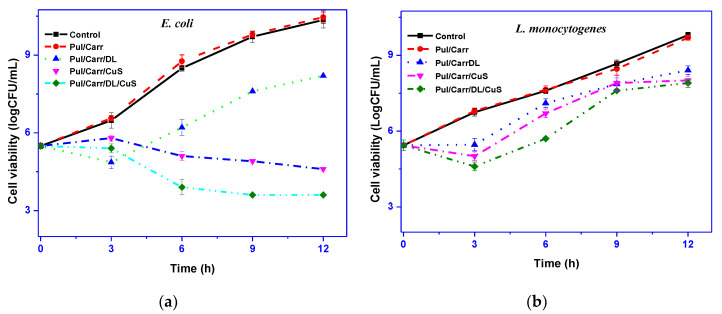
Antimicrobial activity of pullulan/carrageenan-based films against (**a**) *E. coli* and (**b**) *L. monocytogenes*.

The antimicrobial activity of all the film samples is presented in Figure 6. The control and neat polymer film do not display any antimicrobial activity against tested bacteria, whereas the films comprising CuSNP and DL showed some antibacterial activity against tested strains. The antibacterial activity of CuSNP was more effective compared to DL. However, the composite film containing the combined filler displayed a superficial bactericidal effect. It was observed that the activity of CuSNP is more comprehensive and showed a significant reduction in the growth of *E. coli* (about 7 log cycles) after culturing for 12 h compared to the control, whereas, in the case of *L. monocytogenes,* a slight decrease in growth witnessed (about two log cycles). The variation of antimicrobial activity against *E. coli* and *L. monocytogenes* was most probably due to the cell wall structure’s difference. The obtained results suggested that DL showed some antibacterial activity against *E. coli*, but CuSNP exhibited efficient antibacterial activity against *E. coli* and some activity against *L. monocytogenes*. It is previously reported that the CuSNP showed antibacterial activity only at higher content (2%), and in this work, as only 0.5% of CuSNP was used, intense antibacterial action was not observed. Like the current findings, the potent antimicrobial activity of CuSNP-loaded agar, alginate, and carrageenan composite film was reported previously [15,16,25]. The antibacterial activity of CuSNP was not exactly elucidated yet but believed to damage the cell membrane and oxidative damage through the formation of reactive oxygen species [42]. The DL-containing film’s antimicrobial activity was comparatively lower than predictable; however, a similar low antimicrobial activity has been reported previously [22]. In the case of DL, the antibacterial activity is dependent on the content and also on the microbial strain [28]. The antibacterial mechanism of DL relies on the ability to penetrate through the microorganism’s cell wall, which disturbs the cell viability [22]. Overall, the Pul/Carr-based film with moderate antimicrobial action can delay the growth of the microorganism.

## 4. Conclusions

CuSNP and limonene were reinforced to produce a pullulan/carrageenan-based novel composite film. The fillers were compatible and homogeneously spread in the matrix film. The incorporation of fillers meaningfully enhanced (>75%) the UV-light shielding property of the films without greatly losing the transparency (~20%). The mechanical properties (tensile strength, elongation at break, and elastic modulus) of the film are also meaningfully improved (~15%), while the thermal stability remains unaltered. The water vapor barrier property was increased slightly (~10%), and hydrophobicity remains unchanged. The developed film also showed good antimicrobial activity against the pathogenic food microbe, which can be used to control the growth of the microorganism in packaged foods. The fabricated pullulan/carrageenan-based functional film with increased film properties can be used for active food packaging applications. For practical application of the edible active packaging film, additional research is required for mass production of functional fillers, industrial production of functional films using biopolymers, and application of developed films.

## Figures and Tables

**Table 1:** Surface color and transmittance of pullulan/carrageenan-based composite films.

Films	*L*	*a*	*b*	Δ*E*	*WI*	*C*	*YI*	T_280_ (%)	T_660_ (%)
Pul/Carr	91.3 ± 0.1 ^b^	−0.6 ± 0.0 ^b^	5.6 ± 0.1 ^a^	1.6 ± 0.1 ^a^	93.6 ± 0.1 ^c^	5.6 ± 0.1 ^a^	8.8 ± 0.1 ^a^	70.1 ± 1.2 ^b^	88.3 ± 0.4 ^b^
Pul/Carr/DL	91.3 ± 0.0 ^b^	−0.6 ± 0.0 ^b^	5.6 ± 0.1 ^a^	1.6 ± 0.1 ^a^	93.7 ± 0.1 ^c^	5.6 ± 0.1 ^a^	8.7 ± 0.1 ^a^	71.0 ± 1.6 ^b^	88.4 ± 0.7 ^b^
Pul/Carr/CuS	76 ± 0.6 ^a^	−4.9 ± 0.7 ^a^	17.2 ± 0.8 ^c^	20.9 ± 0.8 ^b^	81.5 ± 0.6 ^a^	17.9 ± 0.6 ^c^	32.1 ± 1.8 ^c^	18.5 ± 3.2 ^a^	69.5 ± 1.9 ^a^
Pul/Carr/DL/CuS	76.3 ± 0.5 ^a^	−5.2 ± 0.4 ^a^	15.8 ± 0.8 ^b^	20.2 ± 0.3 ^b^	82.7 ± 0.7 ^b^	16.6 ± 0.7 ^b^	29.6 ± 1.4 ^b^	15.6 ± 2.1 ^a^	68.1 ± 1.4 ^a^

The values are represented as a mean ± standard deviation. In the same column, any two means, followed by the same letter, are not significantly (*p* > 0.05) different from Duncan’s multiple range tests.

**Table 2:** Mechanical properties, WVP, WCA, and MC of pullulan/carrageenan-based films.

Films	Thickness (μm)	TS (MPa)	EB (%)	EM (GPa)	MC (%)	WVP (×10^−9^ g m/m^2^ Pa s)	WCA (deg.)
Pul/Carr	48.3 ± 3.0 ^a^	54.0 ± 6.5 ^a^	2.7 ± 0.3 ^a^	3.4 ± 0.3 ^a^	10.9 ± 0.6 ^a^	1.0 ± 0.1 ^ab^	61.6 ± 4.2 ^a^
Pul/Carr/DL	47.4 ± 1.8 ^a^	54.5 ± 7.3 ^a^	3.0 ± 0.4 ^a^	3.2 ± 0.2 ^a^	11.5 ± 0.3 ^a^	0.93 ± 0.1 ^ab^	64.0 ± 3.2 ^a^
Pul/Carr/CuS	48.8 ± 2.8 ^a^	66.5 ± 6.6 ^b^	4.0 ± 0.3 ^b^	3.4 ± 0.2 ^a^	12.0 ± 1.0 ^a^	1.06 ± 0.1 ^b^	61.7 ± 3.0 ^a^
Pul/Carr/DL/CuS	47.5 ± 3.5 ^a^	70.5 ± 3.2 ^b^	4.1 ± 0.3 ^b^	3.7 ± 0.3 ^b^	11.6 ± 0.3 ^a^	0.87 ± 0.1 ^a^	63.7 ± 2.4 ^a^

The values are represented as a mean ± standard deviation. In the same column, any two means, followed by the same letter, are not significantly (*p* > 0.05) different from Duncan’s multiple range tests.

**Table 3:** Thermogravimetric data of pullulan/carrageenan-based films.

Films	T_onset_/T_end_ (°C)	T_0.5_ (°C)	Char Content (%)	T_max_ (°C)
Pul/Carr	160/445	360	36.2	240
Pul/Carr/DL	160/440	361	37.1	239
Pul/Carr/CuS	159/441	370	38.1	240
Pul/Carr/DL/CuS	159/445	372	38.3	240
